# Thinking about Death Reduces Delay Discounting

**DOI:** 10.1371/journal.pone.0144228

**Published:** 2015-12-02

**Authors:** Nicholas J. Kelley, Brandon J. Schmeichel

**Affiliations:** 1 Northwestern University, Department of Psychology, Evanston, Illinois, United States of America; 2 Texas A&M University, Department of Psychology, College Station, Texas, United States of America; University of New South Wales, AUSTRALIA

## Abstract

The current study tested competing predictions regarding the effect of mortality salience on delay discounting. One prediction, based on evolutionary considerations, was that reminders of death increase the value of the present. Another prediction, based in part on construal level theory, was that reminders of death increase the value of the future. One-hundred eighteen participants thought about personal mortality or a control topic and then completed an inter-temporal choice task pitting the chance to gain $50 now against increasingly attractive rewards three months later. Consistent with the hypothesis inspired by construal theory, participants in the mortality salience condition traded $50 now for $66.67 in three months, whereas participants in the dental pain salience condition required $72.84 in three months in lieu of $50 now. Thus, participants in the mortality salience condition discounted future monetary gains less than other participants, suggesting that thoughts of death may increase the subjective value of the future.

## Thinking about Death Reduces Delay Discounting

“carpe diem, quam minimum credula postero”

“Seize the day, trusting tomorrow as little as possible”

Horace (*Odes* 1.11)

How does awareness of death influence the value one puts on the future? There are two general possibilities. The opening quotation distillates a common sentiment in western culture: Live for today. Death is a stark reminder that the future is not guaranteed, so it is reasonable to believe that thoughts of personal mortality make one even more inclined to value today over the future. On the other hand, people make plans and strive for goals (including more life) that can only be met in the future. These future goals help give meaning to the present and can provide psychological protection against the threat of death. In this sense it seems reasonable to believe that reminders of personal mortality make the future seem even more valuable—a precious commodity in short supply [1]. The current experiment assessed delay discounting to determine the manner in which reminders of death influence the value of the future.

Delay discounting refers to a present-biased valuation of rewards in which individuals discount the value of future rewards in favor of smaller, more immediately-available rewards. Delay discounting rates have implications for a wide range of behaviors that pit short-term gains against long-term investments, including dieting [2], financial planning [3], and investing in public goods [4]. Understanding the psychological processes that make individuals more or less present-focused is a crucial step toward solving problems associated with high delay discounting rates. We reasoned that thoughts of death are likely to influence discount rates and tested two general possibilities. First, thoughts of death may make individuals more future-focused and thus lead to lower discount rates (i.e., valuing the future). Second, thoughts of death may make individuals more present-focused and thus produce higher discount rates (i.e., valuing the present). Research inspired by terror management theory provides evidence for both possibilities.

### Terror Management Theory

According to terror management theory (TMT) [5, 6]), the uniquely human awareness of death, combined with the drive for self-preservation, elicits a potentially paralyzing existential anxiety that influences much of human psychological experience and interpersonal behavior [5, 6]. Several experiments have supported TMT by finding that manipulations to increase the salience of death trigger defensive responses aimed at preventing or reducing potential anxiety [7–10]. Consistent with the hypothesized role of potential anxiety in TMT, individual differences in anxiety-related traits (e.g., neuroticism) and self-esteem have been found to influence the use of defensive responses when mortality is salient [11–13]. The two major defenses against potential death anxiety include bolstering symbolic conceptions of reality that give order, meaning, and stability to life (i.e., worldview defense), and increased striving to live up to cultural standards of value and thereby to attain literal immortality (e.g., life after death) or symbolic immortality (e.g., transcendence of death through one’s achievements or offspring) [7, 14].

Symbolic immortality is thought to be sought via five types of behavior [15]. One route to symbolic immortality is through the biological propagation of one’s genetic line, which may provide a sense that one is connected to the past through one’s parents and to the future through one’s offspring. A second path involves obtaining symbolic immortality through creative pursuits. For example, teaching students, publishing papers, and writing books may allow an academic to feel as though their unique scholarly products will live on after they cease to exist. A third path is to find a sense of connection to a larger universe, which may help one to feel as though one is part of something more permanent than the individual self. A fourth route to symbolic immortality involves the transcendence of the physical self through spiritual attainments. A fifth route may be to lose oneself in intense experiences. Florian and Mikulincer [16] found that high levels of self-reported symbolic immortality are inversely related to death anxiety, and higher self-reported symbolic immorality reduces defensive responses to mortality salience (e.g., more extreme punishments for moral transgressors). Thus, symbolic immortality appears to be a key to reducing death anxiety and the aftereffects of mortality salience.

### Mortality salience and a focus on the future

Desire for immortality provides a clue to a temporal bias that may emerge in response to reminders of death—a focus on the future. Insofar as reminders of death engender a focus on the future, thinking about death should decrease delay discounting rates as individuals become more concerned with what the future holds rather than what they may gain right now.

In addition to evidence for increased immortality striving, theory and research on levels of mental construal also lend indirect support to the idea that mortality salience increases future-oriented concerns. According to construal level theory [17], hypothetical events and future events (e.g., one’s own eventual death) tend to be construed in abstract or high-level terms. This is in contrast to recent or actual events (e.g., the experience of dental pain), which tend to be construed in more concrete, low-level terms [18, 19]. Personal mortality can be seen as a hypothetical future event that looms as an inevitable reality. Insofar as thinking about death promotes more abstract or future-oriented mindsets, reminders of death may increase the value of the future and reduce delay discounting.

Prior evidence supports the assumption that thinking about death promotes abstract, high-level mindsets associated with an orientation toward the future. For example, Landau, Kosloff, and Schmeichel [20] found that mortality salience primes increase the tendency to identify actions at more abstract (versus concrete) levels of identification. More specifically, participants who had (versus had not) been primed with thoughts of death were more likely to construe the act of voting as participating in democracy rather than as pushing buttons on a screen. People who endorse abstract action identifications tend to be less impulsive [18, 19], and experimental manipulations to induce more abstract or high-level construals have been found to reduce impulsivity and improve self-control [21–23]. Given that mortality primes can induce more abstract, high-level construals associated with good self-control, thoughts of death may orient one toward the future and thus reduce delay discounting.

### Mortality salience and a focus on the present

The opposite prediction (i.e., that mortality salience increases preferences for smaller, more immediate rewards) is also plausible and has received support in previous research. For example, Gailliot, Schmeichel, and Baumeister [24] found evidence for poorer self-regulation under mortality salience. Relative to a control condition, contemplating personal mortality caused worse performance on a variety of cognitive challenges, including a Stroop task and word puzzle. Gailliot and colleagues proposed that contemplating death depletes self-regulatory resources, thereby increasing the influence of short-term desires and undermining long-term goal pursuits. Other studies have found similar evidence; mortality salience has been observed to increase risky driving intentions and behaviors [25], gambling [26], and sun tanning [27] for persons who report these behaviors being particularly relevant to their self-esteem. These studies bespeak a preference for immediate versus more delayed gratifications particularly when those gratifications underlie one’s self-esteem.

Evolutionary views of human nature, which include terror management theory, assume the mind is adaptively rational to the extent that it evolved to solve survival-relevant challenges in ancestral environments [28, 29]. It may seem rational to favor larger future rewards over smaller immediate rewards by a simple comparison of magnitudes, but that may not be a decision the human mind was biologically prepared to make. Ancestral environments were often unpredictable, lifespans were shorter, and death was an ever-present threat. Forgoing immediate rewards in such an environment could have carried steep costs. In this view, when death is salient, favoring smaller, more immediate gains may be the more adaptive choice.

Consistent with this perspective, and using life history theory as a guide, Griskevicius, Tybur, Delton, and Robertson [30] found evidence that death primes can increase preferences for smaller, more immediate rewards. More specifically, after reading an article that discussed violence and death in the 21^st^ century, participants preferred smaller immediate rewards over larger, delayed rewards. This pattern was strongest among participants who grew up in resource-scarce environments, which suggests that persons with relatively fast life history strategies value the present over the future when considering threatening circumstances.

Although the studies by Griskevicius and colleagues examined the effects of death-related ideation on delay discounting, the current work differs by following research based on terror management theory to examine the effect of personal morality concerns (rather than broader societal ones) on delay discounting rates. The current study differed from previous studies in another crucial respect. Most of the studies reviewed above included a delay period following the mortality salience induction, because theory and evidence suggest that defensive responses to reminders of death emerge mainly after participants have had the opportunity to suppress or minimize death’s salience [31, 32]. But more proximal responses to reminders of death have been found to emerge immediately after a mortality salience induction. The distinction between proximal versus distal responses to mortality salience is well established, with immediate, proximal responses tending to be more rational relative to the more defensive delayed responses [33, 34]. Vail and colleagues [10] proposed that the proximal response to conscious death thoughts is to shift the mind away from individualistic, present-focused, status-oriented goals (e.g., wealth) toward more abstract, communal goals that can help to build stronger communities and interpersonal relationships. Consistent with this view, Wade-Benzoni, Tost, Hernandez, and Larrick [35] found that a death reminder had the immediate effect of causing participants to donate more money to a charity to help the needy in the future compared to the present.

Because the delay period following mortality salience may involve depleting acts of thought suppression [24], and because research including a delay period has yielded mixed evidence relevant to delay discounting, we elected to omit a delay period after the mortality salience induction in the current study. The absence of a delay period allowed us to investigate the immediate, proximal effects of death thoughts on valuing the present versus the future, free of potential confounds from resource depletion. Thus, we asked participants to ponder their own mortality or a control topic immediately prior to completing a measure of delay discounting.

### The Current Study

We identified two competing hypotheses. One is in accord with previous research on terror management theory and insights from construal level theory and assumes that outcomes that are congruent with a particular mindset would be valued more than outcomes that are incongruent with a particular mindset. Because future events (such as one’s own death) tend to be construed in more abstract or high-level terms, a more delayed reward would be congruent with a high level or abstract mindset. Hence, mortality salience should increase concern for the future and cause people to value future monetary gains more than they otherwise would. The other hypothesis accords well with evolutionary psychology, previous research on terror management theory, and a common sentiment in western culture: Mortality salience should lead people to “seize the day” and cause them to value future monetary gains less than they otherwise would. We conducted an experiment to put these hypotheses to the test.

## Method

### Participants and Procedure

One hundred eighteen undergraduate students (90 women) satisfied a course requirement by participating. Participants completed the study in a classroom and were randomly assigned to either a mortality salience or dental pain condition. Participants ages ranged from 18 to 43 (*M* = 21.19, *SD* = 2.88). Participants were predominantly white (80.51%) and non-Hispanic (88.14%).

#### Ethics Statement

The study was approved by the Institutional Review Board (IRB) at Texas A&M University. A waiver of informed consent was approved by the IRB because the study involved no more than minimal risk to participants. Verbal informed consent was obtained from all participants after they read an information sheet (essentially an informed consent form that does not ask for a signature). If they consented, they were allowed to participate. Therefore, their data reflects documentation of their consent.

#### Statistical power and sample size selection

A recent meta-analysis [9] revealed that the average mortality salience effect is medium to large in size (*r* = 0.35) and that the prototypical mortality salience experiment includes approximately 87 participants (*M* = 87.3, *SD* = 50.8). We elected to collect a larger sample of participants than the average mortality salience study due to concerns about replicability within psychological science. Sampling 118 participants afforded us .76 power to detect a medium-sized (*r* = 0.25) main effect of mortality salience on delay discounting behavior.

### Materials

#### Mortality Salience

Following previous research [5, 12], participants in the *mortality salience condition* (*N* = 60) were prompted to “Please briefly describe the emotions that the thought of your own death arouse in you” and “Jot down, as specifically as you can, what you think will happen to you as you physically die and once you are physically dead.” In the *control condition* (*N* = 58), participants responded to parallel prompts about a painful dental procedure. Immediately following the manipulation participants completed the delay discounting task.

*Delay Discounting*. Participants made hypothetical choices pitting an immediate reward against a delayed but more valuable reward. Specifically, participants made a series of choices between receiving $50 now versus receiving other dollar values three months later, starting with $50 and increasing in $5 increments up to $100. The indifference point was the dollar value at which participants switched from preferring the fixed immediate amount ($50) to preferring the delayed amount. If the participant never switched, the indifference point was coded as $105. Thus, $50 now was pitted against increasing possible future rewards. The delay discounting rate was quantified following the recommendations of Weber et al. [36], whereby a value of 1 indicates no discounting and smaller values indicate greater discounting of future rewards.

## Results

Mortality salience (*M* = 0.33, *SD* = 0.28) reduced delay discounting rates compared to dental pain salience (*M* = 0.46, *SD* = 0.26), *t* (116) = 2.44, *p* = .02, *d* = 0.49, 95% CI (.44, 0.53). In monetary terms, the observed indifference points revealed that participants in the mortality salience condition would trade $50 now for $66.67 in three months, whereas participants in the dental pain salience condition would give up the same $50 for $72.84 in three months. Put differently, participants in the mortality salience condition discounted future monetary gains less than did other participants, suggesting that they valued the future more (S1 File).

## Discussion

The current study was the first to ask how contemplating personal mortality affects delay discounting. Consistent with theorizing based upon construal level theory, the results revealed a greater preference for future rewards (i.e., a decreased discount rate) following mortality salience versus dental pain salience. Simply put, mortality concerns appear to cause people to value the future more.

The results of the current experiment are consistent with prior evidence of proximal responses to mortality salience. Recall that proximal responses are immediate responses to the conscious consideration of mortality. Proximal responses, in contrast to more distal responses, are often rational, future-oriented, and intended to terminate bad habits that may hasten death [10, 37]. Likewise, the current experiment found that mortality salience had the immediate effect of causing participants to discount future monetary gains less (value future monetary gains more), relative to dental pain salience. Given that delay of gratification assists successful goal pursuit and contributes to several desirable outcomes in life (including higher levels of educational attainment, income, fidelity, and lower body mass index; e.g., [38]; see also [39]), the observed evidence for reduced delay discounting rates represents a seemingly rational response to reminders of death.

As Pyszczynski and colleagues [36] noted, however, thoughts of death are often buried deep within the mind, away from conscious awareness. Accordingly, most TMT studies have distracted participants after mortality salience or allowed participants to suppress their death-related cognitions before assessing the dependent measure of interest. Relative to proximal or immediate responses to mortality salience, these more distal responses appear irrational and defensive. For example, out-group derogation, harsh punishments for norm violators, and impulsive behaviors are established distal responses to mortality salience. The process of suppressing or burying death-related cognitions can deplete cognitive resources, which may help fuel distal defenses [23, 40]. By avoiding the potentially depleting effects of death-related thought suppression, the current findings attest to a proximal response to thoughts of death—increasing the value of future (versus present) monetary gains. Proximal responses to mortality salience are much less investigated than distal responses in the TMT literature, and the same is true of the potential positive consequences of mortality salience. By examining an immediate and positive consequence of mortality salience, the current results help to broaden understanding of mortality salience effects. However, the current research did not assess more delayed or distal responses to mortality salience, so additional research is needed to determine whether delay discounting rates are reduced, increased, or stable when thoughts of death have receded from conscious awareness.

How can people become motivated to eat well, exercise more, and save more money for the future? Similarly, how can society deal with the obesity epidemic, large scale debt, and climate change? Terror management and construal level theories may provide a starting point. Vail and colleagues [10] suggested that when mortality is in focal awareness, more intrinsically meaningful goals, which tend to be construed in more abstract and future-oriented, become activated to enable goal pursuits that help to forestall the grim inevitability of death. By contrast, more materialistic, extrinsic goals, which tend to be construed as more concrete and present-oriented, are avoided or their importance is downplayed when death is in focal awareness. It may be the case that conscious mortality awareness activates intrinsic goal pursuit and minimizes the importance of extrinsic goals because the future is seen as more valuable. Put differently, mortality salience may increase the value of the future, which in turn may activate relatively healthy, future-focused goal pursuits.

Moreover, although death is omnipresent in western cultures, persons in these cultures suffer from a variety of problems associated with high discount rates, including obesity, large scale debt, and the threat of climate change. How can these troubling patterns persist if, as we have observed, reminders of death reduce delay discounting? We propose that, although the proximal response to mortality salience is to care and plan for the future, this tendency fades when mortality is not at the center of awareness. When death recedes from focus to linger on the fringes of awareness, poor self-control and selfish decision making seem to come to the fore [24].

### Terror Management and Life History Theories

Whereas the current experiment found a reduction in delay discounting among persons who had just pondered their own mortality, previous research inspired by life history theory found that thoughts of a treacherous, death-laden future led persons lower in SES to increase delay discounting [30]. How can thoughts of death lead to both more and less delay discounting? We think one crucial difference between the two findings is that the current experiment tested the effect of *personal* mortality on delay discounting, whereas the research based on life history theory focused on the effects of more *general* mortality concerns. Although TMT and life history theory both recognize the importance of mortality concerns in motivating human behavior, life history theory emphasizes the importance of resource scarcity and other environmental factors that helped to shape human evolution, whereas TMT focuses on more micro-level interpersonal and intrapersonal processes.

It may be that the group-level threat of war and death featured in previous research on life history theory and delay discounting [30] triggered a greater motivation for survival in the present (hence more discounting) relative to the typical mortality salience induction. Additionally, it could also be the case that the typical mortality salience prime induces a more abstract or future-oriented mindset relative to the war-based life history prime. Either of these possibilities may account for the apparent differences between the current findings and prior findings [30]. It is also important to acknowledge that participants in the current study showed reduced delay discounting under mortality salience, similar to higher SES participants in the study by Griskevicius and colleagues [30]. We did not assess participants’ SES in the current study. Future research exploring differential reactions to personal versus societal mortality salience as well as the role of SES in responses to mortality salience seems warranted.

### Limitations and Alternative Explanations

In the current research we used dental pain as a control condition, as is typical in TMT research [9]. The dental pain control condition is considered a useful comparison for the mortality salience condition because pain, like death, is an aversive stimulus. Hence, insofar as thinking about death and thinking about dental pain have different effects, these effects are seemingly not attributable to pondering an aversive topic. But we did not include a neutral control condition in the current study—in both the dental pain condition and the mortality salience condition participants pondered an aversive topic. We therefore cannot rule out the possibility that pondering dental pain influenced delay discounting. For example, delay discounting may have been lower in the mortality salience condition than in the dental pain condition because pondering dental pain made persons more present focused. Indeed, some evidence suggests that the experience of pain may lead persons to focus on the present. Specifically, Bastian and colleagues [41] found that the experience of pain led participants to choose delicious desserts over other, less enticing rewards (e.g., pens). Insofar as desserts are considered immediate rewards, this evidence suggests that the experience of pain may lead persons to focus on the present or on more immediate rewards. Whether merely thinking about pain has the same effects as experiencing pain remains an open question. Nonetheless, future research should explore how death and pain influence discounting behavior by comparing them both to the effects of pondering a neutral topic.

The current research included one experiment assessing the effect of mortality salience on delay discounting behavior. This single study suffers from at least two important limitations. First, given recent concerns about the replicability of findings in social and personality psychology, future experiments should attempt to replicate the current findings. Although the current study was adequately powered to detect a medium-sized effect of mortality salience, as discussed above, attempts to replicate the current results will allow researchers to understand the true size of the effect of morality salience on delay discounting. Second, the current experiment focused on proximal responses to mortality salience (i.e., responses measured immediately after the mortality salience induction) and thus cannot speak to the effect of mortality salience on delay discounting as a distal or delayed response to mortality salience. Would a delay after the mortality salience induction lead to a focus on the future, a focus on the present, or no effect at all? As reviewed previously, we suspect that after participants have had the opportunity to suppress or otherwise push death thoughts out of focal awareness that thinking about death would lead to a focus on the present. Future research is needed to test this hypothesis.

### Where is the “terror” in terror management?

Last, the current findings may have implications for understanding the role of emotions in terror management. To the surprise of some theorists [42], standard mortality salience inductions often fail to change participants self-reported emotional states, presumably because death anxieties are suppressed, sublimated, or otherwise defused. But it may be that standard mortality salience inductions simply do not induce a strong emotional response. Emotional and motivational systems are strongly tuned to proximal goals and events [43]. Death, by contrast, is a difficult to comprehend, abstract, and temporally distant future event for most individuals. Thus, death may prime an abstract, future-oriented mindset that is not conducive to intense emotion, but is conducive to valuing the future.

## Supporting Information

S1 File Supporting SPSS Data File. Mortality salience reduced delay discounting rates compared to dental pain salience.
(SAV)
